# Rare Case of Sclerosing Epithelioid Fibrosarcoma of the Thoracic Spine

**DOI:** 10.1155/cro/3645053

**Published:** 2026-04-01

**Authors:** Michael Marcinko, Shaan Sadhwani, Brendan Sweeney, Timothy Edwards, Tyson Maugle

**Affiliations:** ^1^ Department of Orthopaedic Surgery, UPMC Community Osteopathic, Harrisburg, Pennsylvania, USA; ^2^ Orthopedic Institute of Pennsylvania, Harrisburg, Pennsylvania, USA

## Abstract

**Background:**

Sclerosing epithelioid fibrosarcoma (SEF) is a rare sarcomatous tumor that presents infrequently as an osseous lesion of the spine. To our knowledge, few case reports exist regarding primary lesions of the lumbar spine, with only one report indicating the thoracic spine as a primary site.

**Case Description:**

We present a case of a 32‐year‐old female presenting with primary osseous SEF with complaints of axial and right‐sided thoracic back pain for 4 months. Imaging of the thoracic spine revealed a mass at T5 with vertebra plana and central spinal cord compression at T5–T6 without significant expansion of mass to soft tissues or adjacent vertebral levels. We performed a T5 corpectomy with posterior spinal instrumented fusion from T3 to T7. Pathologic and immunohistochemical studies confirmed the diagnosis of SEF. One‐year follow‐up revealed no recurrence of disease and significant pain relief without neurologic dysfunction.

**Conclusions:**

In light of the extent of the vertebral body involvement, the severity of cord compression, and the patient′s associated neurologic symptoms, a T5 corpectomy with T3–T7 posterior fusion was performed. Given the nature of the spine, complete resection of these tumors is usually impossible, and adjuvant radiation or chemotherapy may be necessary for resolution of disease.

## 1. Introduction

Primary spine tumors are devastating diagnoses cited by the American Cancer Society to account for approximately 5% of the roughly 6000 primary bone tumor diagnoses each year. This equates to upwards of 8.5 per 100,000 people with a new diagnosis each year [1]. The most common primary malignant tumors of the spine are Ewing sarcoma, chordoma, osteosarcoma, and chondrosarcoma. Although these are the most common primary tumors of the spine, this is not an exhaustive list as was seen in the case we present in this report. There exists a dearth of information on the rarer tumor diagnoses and how to definitively manage them.

In this case report we present a 32‐year‐old female with several months of thoracic back pain and weight loss with no past medical history of neoplasm who subsequently was diagnosed with sclerosing epithelioid fibrosarcoma (SEF) of the thoracic spine confirmed on biopsy. SEF was first described in 1995 by Meis‐Kindbloom et al., but remains an extremely rare diagnosis with one report demonstrating 51 cases over a 22‐year period at a single institute [2, 3]. Although the exact incidence is lacking in the literature, the diagnosis is more commonly made in the lumbar spine and pelvic girdle as a deep‐seated intramuscular lesion. However, these tumors can rarely present as primary osseous/intraosseous lesions. Furthermore, when these lesions are osseous in origin, they are rarely found in the thoracic spine and are more commonly found in the lumbar spine [4]. To our knowledge, few case series and case reports describing this disease as an osseous lesion in the spine exist and only one other case report of a thoracic spine lesion as the primary site of SEF [4–8].

We present a case of a primary SEF of the thoracic spine and explore the operative and clinical considerations associated with this peculiar pathology. Given the extent of the vertebral body involvement, the severity of cord compression, and the patient′s associated neurologic symptoms, a T5 corpectomy with T3–T7 posterior fusion was performed. This report has been approved by our institution′s institutional review board. The patient was informed that the details about the case would be submitted for publication, and written permission was obtained.

## 2. Case History

A 32‐year‐old female presented with 4 months of upper thoracic pain localized to the right side of her upper rib cage. She reported a past medical history of polycystic ovarian syndrome, gestational diabetes, and chiropractic care in the past. She denied any significant inciting event or trauma. Initial workup involved a cervical MRI for cervical radiculopathy and subsequent cervical epidural steroid injection, which provided minimal relief. At the time of initial presentation, she denied radiculopathy, upper motor neuron signs in her upper and lower extremities, saddle anesthesia, or bowel/bladder dysfunction. On physical exam, the patient was neurovascularly intact with excellent strength in her upper and lower extremities. Lumbar flexion and extension reproduced the back pain, and she was mildly tender in the right upper paraspinal thoracic musculature. She did have numbness through the left lower extremity in the left thigh, calf, and foot.

**Figure 1:**
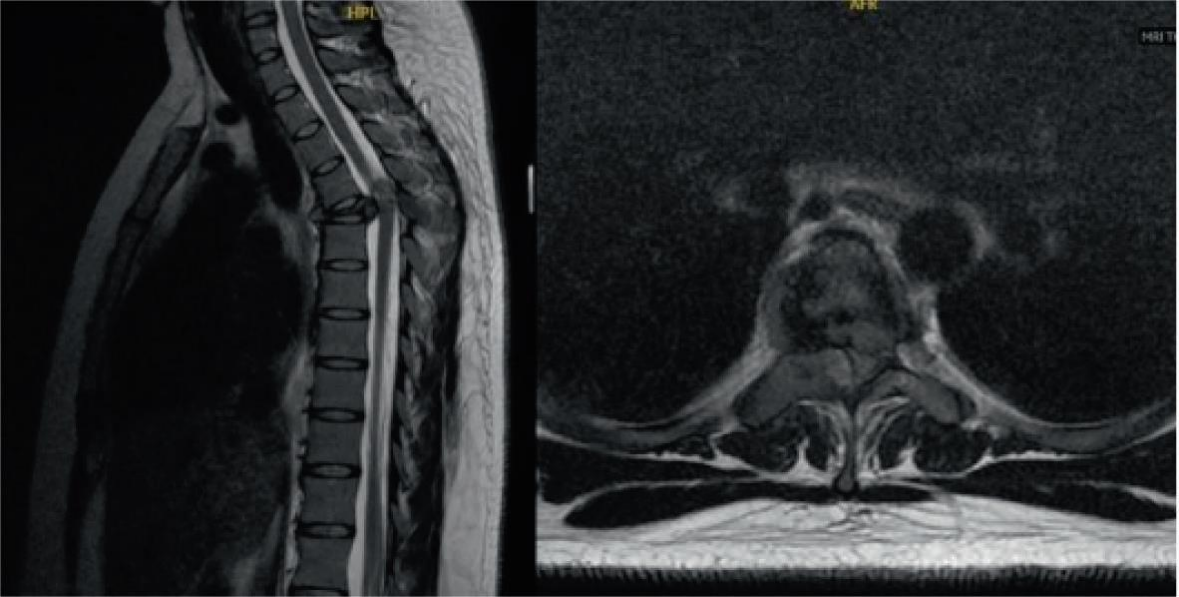
MRI imaging showing (a) axial T2 at the level of T5 and (b) sagittal T2 at the level of T5.

An MRI of the thoracic spine was obtained that showed thoracic vertebral plana at T5 with bony retropulsion and severe canal stenosis. (Figure 1) Hyper‐expansion of the right pedicle and lamina at T5 was seen. She underwent further workup for malignancy by the hematology/oncology team with computed tomography (CT) scan, labs, and biopsy by interventional radiology. CT of the chest, abdomen, and pelvis was unremarkable for other lesions or pathologic processes. Laboratory workup was also unremarkable and showed no renal insufficiency, hypercalcemia, or M‐spike to indicate myelopathy. Given these findings and her severe stenosis, progressive pain and numbness, she elected to undergo surgical resection prior to final pathology result.

After thorough imaging review, we elected to perform a thoracic bilateral laminectomy of T5–T6 with T5 corpectomy and posterior fusion of T3–T7. After general anesthesia, Foley, Mayfield holder, and neuromonitoring leads placement, the patient was placed in the prone position. She was noted to have a mild correction of her sagittal deformity just with positioning in the prone position. The thoracolumbar spine was then thoroughly prepped and draped in normal sterile fashion. Skin incision was made over the T5 level and deep dissection was performed across the facet joints and transverse processes of T3–T7. We first performed pedicle screw instrumentation for T3–T7. Upon pedicle cannulation, a ball‐tip probe confirmed no evidence of pedicle breach. Each of the screws was tapped with a 4‐mm tap. We placed 5.0‐mm pedicle screws bilaterally at T3, T4, T6, and T7, and an intraoperative CT scan was obtained to confirm proper placement of each of the pedicle screws. We then placed a temporary rod spanning from T3–T7 on the left to allow for corpectomy on the right. Laminectomy was then performed bilaterally at T5–T6 using a Leksell rongeur and a 3‐mm matchstick bur and completed using a 3‐mm Kerrison rongeur. We extended the laminectomy laterally to include bilateral partial facetectomies and foraminotomies at T5–T6. A complete facetectomy was performed on the right at T5–T6 to allow for the planned corpectomy with a small portion of the T5 rib to allow for trajectory into the vertebral body. The pedicle tract into T5 was then opened using a pedicle reamer. The T5 nerve root was then identified and tracked laterally, in which there was redundancy noted through the T5 nerve root. This allowed for excellent exposure from the T4–T5 disc space down to the T5–T6 disc space.

**Figure 2:**
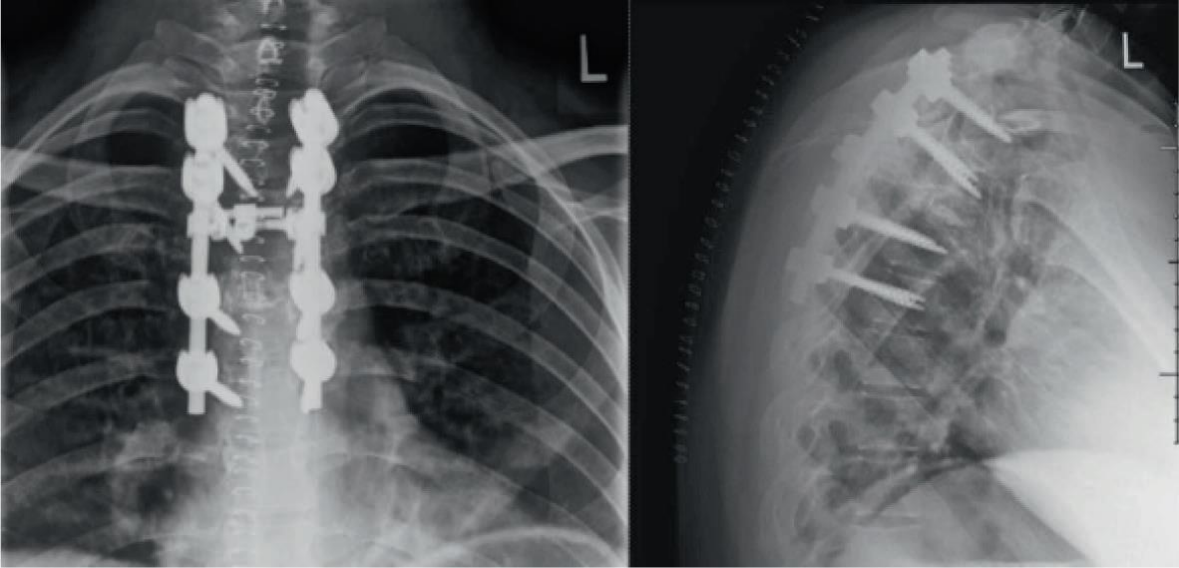
Initial postoperative imaging showing T5 corpectomy with T3–T7 posterior fusion.

A combination of osteotomes, reverse angle curettes, and pituitary rongeurs was then used to complete a corpectomy at T5. We removed as much of the disc and as much of the vertebral body as possible with thorough curettage. Samples were sent for pathology. Frequent neuromonitoring signals were checked, and there were no changes from baseline in the lower extremities. We then moved on to cage placement in which we found that the space between the T4 and T6 endplate was too small for a titanium expandable cage. Therefore, an 11‐mm polyether ether ketone (PEEK) implant with parallel endplates was implanted with the aid of lateral fluoroscopy. The temporary rod placed on the left was then released to allow for compression of the T4 and T6 vertebral bodies against the corpectomy cage. The cage spanned from T4 to T6. This allowed for significant improvement in sagittal alignment. The temporary rod was then tightened again. Additional allograft was then placed along the periphery of the corpectomy cage spanning from T4 to T6. A 5.5‐mm titanium rod was then bent into proper position and spanned from T3 to T7 on the right. This was locked into position into each of the pedicle screws from T3 to T7. The facet joints and transverse processes were then decorticated from T3 to T7. A combination of local autograft and allograft was then placed along the facet joints and lateral gutters spanning from T3 to T7 to allow for arthrodesis. A crosslink was used across the middle of the construct. She was then thoroughly irrigated, a drain was placed, and the wound was closed in a layered fashion. She maintained good lower extremity signals throughout the procedure. Postoperative imaging initially is seen in Figures 2 and 3. She had an optimal postoperative course in which she cleared physical therapy by Day 2, had her drain removed, and was discharged on postoperative Day 3.

Figure 3Representative immunohistochemistry slides demonstrating a tumor with plasmacytoid and focal spindling appearance, eventual diagnosis of EWSR1::CREB3L1 rearranged sarcoma, most compatible with SEF. Immunostains show focal weak positivity for MUM1, negative for CD138, kappa and lambda light chains, AE1/3, CD3, CD 20, S100, SOX10, Melan A, and HMB45 (a,b). Additional stains for were performed. The tumor cells are weakly positive for SMA, with a Ki67 proliferation rate estimated 10%–15%, and negative for desmin, CD10, CD34, Cam 5.2, ER, PR, synaptophysin, Pax8, SATB2, CD 45, and CD 43 (c,d).

**Figure:**
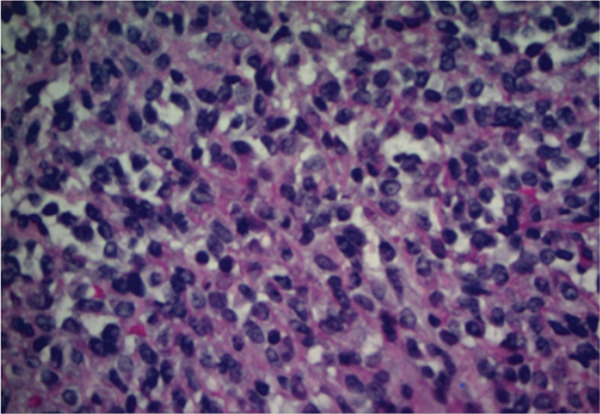
(a)

**Figure:**
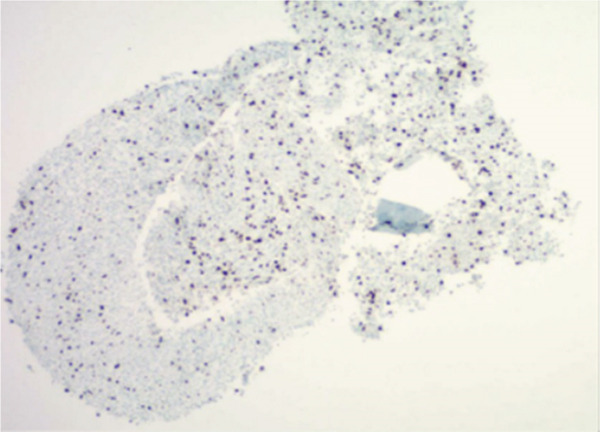
(b)

**Figure:**
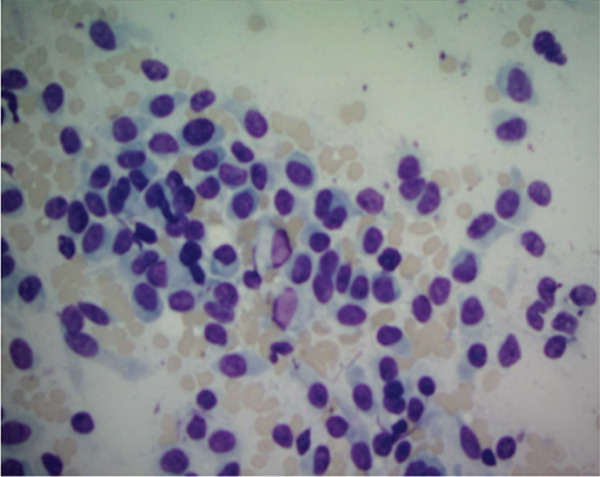
(c)

**Figure:**
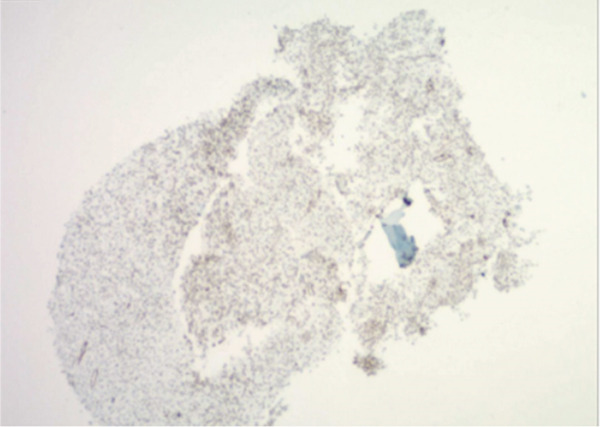
(d)

Pathology from lesional biopsy was concerning for spindle cell neoplasm; however, per the pathology report, this tumor demonstrated plasmacytoid and focal spindling appearance, weakly positive for MUM1 and SMA, low to moderate proliferation rate by Ki 67 (10%–15%), negative for epithelial markers (AE1/3 and Cam 5.2), plasma cell and other hematopoietic cell markers (CD 45, CD 43, CD138, kappa and lambda light chains, CD3, and CD 20), neuroendocrine markers (synaptophysin, CD56 by flow cytometry), endometrial stroma cell markers (CD10, ER, and PR), osteoblastic marker (SATB2), and melanoma markers (S100, SOX10, Melan A, and HMB45). The differentials included sarcoma and some borderline sarcomatous neoplasm (Figure 3). Given these findings, the pathology was sent to a tertiary care center, in which the lesion was then found to be the rare pathology of SEF of the thorax.

**Figure 4:**
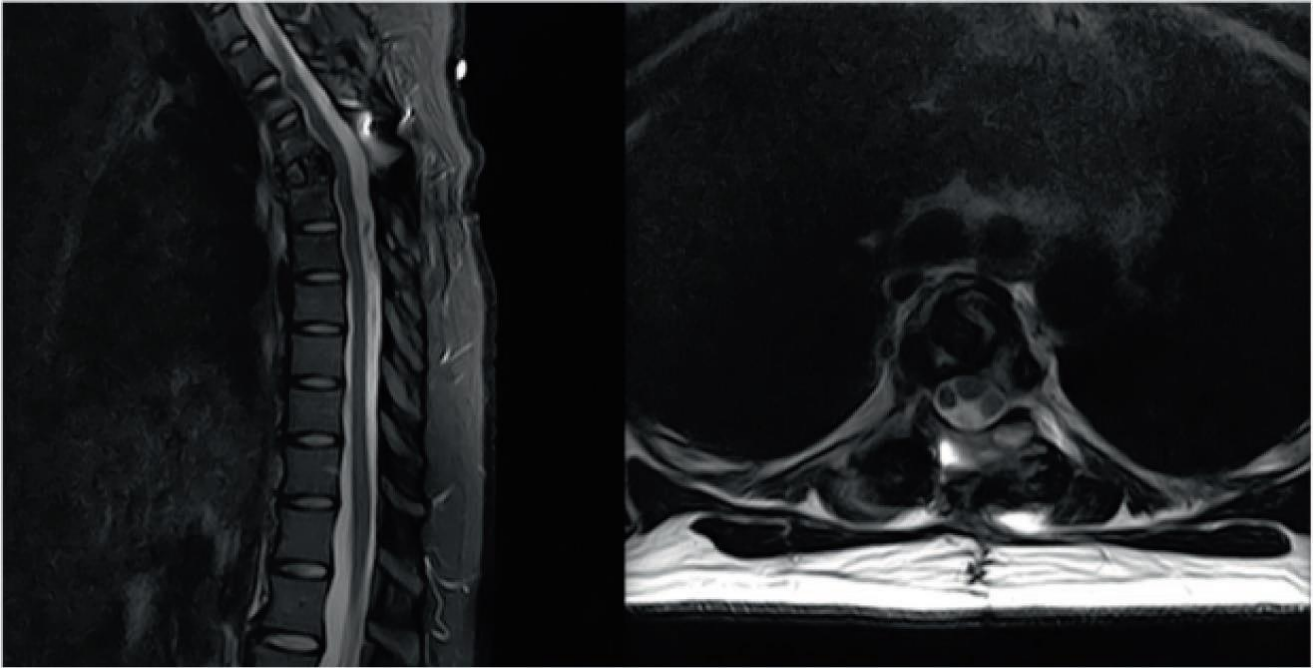
Eight‐month follow‐up MRI demonstrating decompression after T5 corpectomy with T3–T7 posterior fusion.

**Figure 5:**
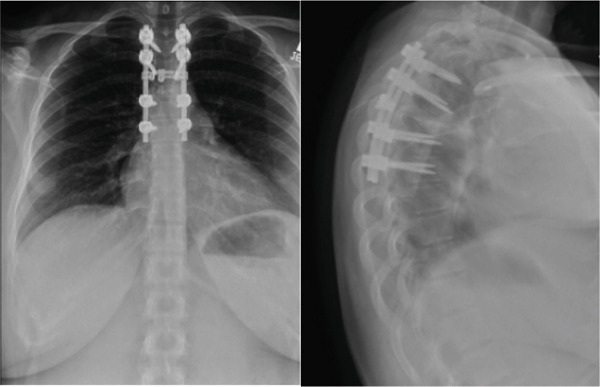
One‐year follow‐up postoperative imaging showing T5 corpectomy with T3–T7 posterior fusion.

Postoperatively, the patient underwent adjuvant radiation per the radiology–oncology team, in which 43.75 gray (Gy) in 25 fractions to the op bed with a 52.5 Gy in 25 fractions boost to the high‐risk area was delivered over 1 month. Her postoperative course was otherwise unremarkable, and at the most recent follow‐up of 1 year, she denied significant pain in her upper back and her left lower extremity numbness had resolved. The only residual symptoms reported were evidence of numbness at the upper part of her prior incision. She was disease‐free on the most recent surveillance CT and was back to her normal activities of daily living. The most recent Xray at 1 year (Figure 4) and MRI at the 8‐month follow‐up (Figure 5) showed continued resolution, no cord compression, and maintained spinal alignment. Remarkably, she is currently planning to have a second child.

## 3. Discussion

We present a case of primary SEF of the thoracic spine, for which the patient underwent T5 corpectomy with T3–T7 posterior instrumentation due to associated cord compression and symptomology. SEF was initially described in 1987 as a low‐grade fibromyxoid sarcoma (LGFMS) distinguished by a swirling whorled growth pattern and cells arranged in sheets that were found in soft tissue of women in their late twenties [9]. In 1995, these findings became a distinct subtype of fibrosarcoma, now known as SEF [2]. Currently, literature describes SEF as a rare and aggressive soft tissue sarcoma with high incidences of local recurrence, metastasis, and overall mortality rate. This sarcoma subtype commonly affects middle‐aged adults with a median age of 45 and has a propensity to affect a wide array of soft tissue/osseous structures. Although spinal involvement is uncommon, the lumbar vertebrae are the most frequently affected. Frequently, SEF metastasizes to the pulmonary system but osseous metastases are common [3]. Primary osseous SEF is even less frequent, with only single institution case reports describing the clinical course [10].

Because of its infrequency, SEF is often difficult to diagnose, especially in primary bone SEF, as it shares morphological and histologic characteristics with LGFMS. Immunohistochemical workup is also difficult, secondary to the overlap between SEF and similar pathologies. MUCIN 4 (MUC 4) is a glycoprotein that is sensitive in SEF (78%), however it is also expressed in LGFMS (99%–100%) [3]. Although LGFMS and SEF have a similar genetic makeup, SEF exhibits a far more aggressive behavior with higher rates of recurrence and metastasis. Thus, molecular studies are often relied on in the workup of these tumors. SEF is characterized by expression of gene EWSR1‐CREB3L1 and EWSR1‐CREB3L2 that differentiates SEF from LGFMS, which expresses FUS‐CREB3L1 [11]. It is important that this differentiation is made between LGFMS and SEF, as SEF is malignant, recurring locally in more than half of the patients and with a metastasis rate of 40%–80%, so it should be treated more aggressively [11]

Surgical resection is the mainstay of management in spinal SEF to provide local control. Previous reports have described resection techniques such as en bloc resection versus corpectomy [7, 12]. En bloc resection would theoretically help minimize local recurrence; however, it has not been shown to impact overall survival and carries greater morbidity risk. Our patient presented with significant tumor burden of the T5 vertebrae. A CT of the chest, abdomen, and pelvis demonstrated vertebrae plana with significant retropulsion into the spinal canal causing severe canal stenosis. Given the extensive tumor burden and worsening neurologic status, we elected to perform laminectomies of levels T5–T6 to provide decompression of the neural elements. We then proceeded to complete the T5 corpectomy meticulously with oncologic principles in mind. Although en bloc resection has theoretical benefits of providing tumor free margins, we elected to perform a corpectomy given the greater morbidity with a high likelyhood to damage of neurovascular structures with en bloc resection.

Typically, a course of adjuvant radiation is warranted to minimize the risk of recurrence and metastasis. The patient we presented did undergo adjuvant radiation per the radiology–oncology team, in which 43.75 Gy in 25 fractions to the op bed with a 52.5 Gy in 25 fractions boost to the high‐risk area was delivered over 1 month as recommended by radiation oncology. Concurrent chemotherapy has limited evidence for the treatment of SEF [13]. Studies with conventional chemotherapeutic agents including doxorubicin, methotrexate, and cisplatin have shown no significant benefit [14]. Given the infrequency of SEF cases, an established treatment algorithm has not been proven. Future research is needed through collaborations with sarcoma specialists to provide a sizeable cohort to compare treatment modalities.

## 4. Conclusions

We report a case of a primary thoracic SEF and our strategy for management. The patient underwent an eventual thoracic bilateral laminectomy of T5–T6 with T5 corpectomy and posterior fusion of T3–T7 with postoperative adjuvant radiation. At 1‐year follow‐up, she remains disease‐free and able to return to her normal daily life with minimal residual symptoms. Given the paucity of data regarding this pathology in the setting of the thoracic spine, we believe this report provides a significant addition to the existing literature by reporting our experience with this extremely rare pathology.

## Funding

No funding was received for this manuscript.

## Disclosure

A preprint has previously been published in [15].

## Ethics Statement

Ethical approval for this study was obtained from the University of Pittsburgh Medical Center, Pittsburgh, Pennsylvania (23E007).

## Consent

No written consent has been obtained from the patients as there is no patient identifiable data included in this case report/series.

## Conflicts of Interest

The authors declare no conflicts of interest.

## Data Availability

Data sharing is not applicable to this article as no datasets were generated or analyzed during the current study.
